# m6A methylation of circKPNA2 promotes colorectal carcinogenesis by activating the RIN1-Ras pathway

**DOI:** 10.1080/15592294.2026.2672219

**Published:** 2026-06-19

**Authors:** Jinhai Tian, Zongying Jiang, Qi Huang, Jia Wang, Yaqin He

**Affiliations:** a General Hospital of Ningxia Medcal University, Yinchuan, China; b Institute of Medical Sciences, General Hospital of Ningxia Medical University, Yinchuan, China; c Department of Pathology, General Hospital of Ningxia Medical University, Yinchuan, China

**Keywords:** Colorectal cancer, circKPNA2, METTL3, RIN1, Ras signaling pathway

## Abstract

Colorectal cancer (CRC) is a prevalent and highly lethal malignancy. However, the molecular mechanisms underlying its progression remain incompletely understood. Therefore, this study aimed to investigate the role and mechanisms of circKPNA2 in CRC progression. We used various techniques, including RNA sequencing, functional assays, RNA pull-down, and mass spectrometry, to examine the expression and function of circKPNA2. Our findings indicate that circKPNA2 is significantly upregulated in CRC tissues and cell lines. It contributes to tumour growth by promoting cell proliferation, migration, and invasion. We found that circKPNA2 specifically binds to the RIN1 protein and increases its levels, thereby activating the Ras signalling pathway and facilitating CRC progression. Additionally, we identified that METTL3 regulates the expression of circKPNA2 through N6-methyladenosine (m^6^A) methylation, which in turn affects CRC cell proliferation, migration, and invasion. These results deepen our understanding of CRC biology and underscore the potential of targeting circKPNA2 as both a therapeutic target and a diagnostic biomarker to improve CRC diagnosis and treatment.

## Introduction

Colorectal cancer (CRC) is a common tumour with high incidence and mortality [1,2]. Its complex molecular mechanisms have not yet been fully elucidated. This study investigates the mechanisms of CRC progression, with a particular focus on the role of circular RNA (circRNA) as a regulatory molecule. In recent years, increasing evidence has shown that circRNA, as a novel regulatory molecule, plays an important role in tumorigenesis and tumour development [3,4]. However, research on the specific mechanisms of circRNAs in CRC remains insufficient. Therefore, further exploration of its functions and molecular mechanisms is necessary to provide a theoretical basis for targeted molecular therapy [5,6].

CircRNA can act as a ‘molecular sponge’ that sequesters miRNA, alleviating miRNA-mediated suppression of target genes, thereby regulating tumour-related gene expression networks [7,8]. This is the well-characterized mechanism of action for circRNA in vivo. CircRNA can also bind to specific proteins, forming protein complexes to regulate downstream signalling pathways, affecting the survival, proliferation, and metastasis of tumour cells. In CRC, it has been found that circGPRC5A promotes tumour growth and metastasis by binding to UBA1 protein, stabilizing PPP1CA protein, and activating Hippo signalling pathway-related factors [9]. Additionally, in recent years, circRNA has received increasing attention for N6-methyladenosine (m^6^A) modification, which plays an important role in regulating circRNA synthesis and stability. For example, the methyltransferase METTL3 enhances the stability of circRUSC2 through m^6^A modification. Downregulation of METTL3 accelerates circRUSC2 degradation, thereby relieving the suppression of miR-661 mediated by circRUSC2 [10]. This leads to downregulation of the tumour suppressor gene TUSC2 and promotes CRC progression. Furthermore, METTL3 binds to circMVP in the nucleus and enhances its stability via m^6^A modification. This upregulates oncogenic circMVP expression, which promotes β-catenin mRNA levels and translation. Consequently, the immune checkpoint B7-H3 expression is upregulated, inhibiting anti-tumour immunity [11].

Although circRNAs have been implicated in various tumours, research on circKPNA2 remains limited. CircKPNA2 May regulate the Ras signalling pathway by modulating RIN1 expression. In this study, we employed RNA sequencing, cell functional assays, cell functional assays, RNA pull-down, and mass spectrometry to comprehensively analyse circKPNA2 expression and its roles in promoting proliferation and invasion in CRC. Furthermore, we found that METTL3-mediated m^6^A methylation contributes to the high expression of circKPNA2 in CRC cells. Collectively, our findings on circKPNA2 provides new insights into the molecular mechanisms underlying CRC progression and offers potential targets for CRC targeted therapy.

## Materials and methods

### Patient selection, tissue collection, and ethical approval

We collected tumour tissues and adjacent normal tissues from 50 patients diagnosed with CRC who underwent surgery. All samples were obtained at the General Hospital of Ningxia Medical University (Yinchuan, China) between July 2021 and March 2024. Patients included in this study had not received chemotherapy or radiotherapy before surgery and had no other infectious diseases or malignancies. Furthermore, all patients were informed about the study, and provided written informed consent. The experimental protocol was approved by the Ethics Committee of Ningxia Medical University (Ethics approval number: 2020–752), all procedures were conducted in accordance with the Declaration of Helsinki.

### CircRNA sequencing and data analysis

We collected the CRC tissue samples and adjacent normal tissues from five patients. Total RNA was then extracted using TRIzol reagent. RNA purity was assessed using a NanoDrop spectrophotometer (Thermo Fisher Scientific), with an OD260/280 ratio between 1.8 and 2.0. We constructed a circRNA library and outsourced high-throughput sequencing to Biomarker Company (Beijing, China) using the Illumina NovaSeq 6000 platform to obtain circRNA expression profiles. Data analysis was performed using DESeq2 software to identify differentially expressed circRNAs. Screening criteria were defined as |log_2_ fold change (FC)| >1 and *p* < 0.05. KEGG pathway enrichment analysis was then conducted for the host genes of these circRNAs. Fisher’s exact test was used to calculate *p*-values, and pathways with *p* < 0.05 were considered significantly enriched.

### Cell culture

Normal intestinal epithelial cells(NCM460) and CRC cell lines(HT29, HCT116, and SW620) were purchased from ATCC Cell Bank (Shanghai, China). These cell lines were maintained in the laboratory of the General Hospital of Ningxia Medical University. They were routinely cultured in RPMI-1640, McCoy 5A, and DMEM media, each supplemented with 10% foetal bovine serum (Gibco) and 1% penicillin-streptomycin; cultures were incubated at 37°C with 5% CO_2_.

### Expression abundance detection

Total RNA was extracted from CRC tissues and cell lines using TRIzol reagent (Invitrogen). cDNA was synthesized with the PrimeScript^TM^ RT reagent kit, and qRT-PCR was performed using TB Green® Premix Ex Taq™ (Takara) on a Roche 480 LightCycler® PCR instrument. Specific primers for circKPNA2 were designed to target its back-splice junction. Each sample was analysed in triplicate, and expression levels were calculated using the 2^− ΔΔCt^ method. All primers used in this study are listed in Table 1.

**Table1.** Sequences of primers/probes used for qRT-PCR, FISH or RNA pull-down.

Gene	Primer/Probe	sequence
GAPDH	Primer	**Forward** -GTTCGTCATGGGTGTGAACC
		Reverse -CATCCACAGTCTTCTGGGTG
METTL3	Primer	**Forward** -CAAGGCTTCAACCAGGGTCT
		Reverse -ACTCAATCTTGCGAGTGCCA
circKPNA2	Primer	**Forward** -TACTCAAGCTGCCAGCTTTCT
		Reverse -GGGCAGCTGGTGTATTAGCA
U6	Primer	**Forward** -CTCGCTTCGGCAGCACA
		Reverse -AACGCTTCACGAATTTGCGT
RIN1	Primer	**Forward** -GAAGCTGCTGTCGCCTAAGA
		Reverse -AGGCTTGAGCACAGAGCAAT
circKPNA2	Probe (FISH)	GAGAAAGCTGGCAGCT+TGAGTAGCT
	Probe	GGTTATGAGACAAAGGGAGAAAGCTGGCAGCTTGAGTAGC
	(polldown)	NC-CCAATACTCTGTTTCCCTCTTTCGACCGTCGAACTCATCG

### Subcellular localization analysis

Fluorescence in situ hybridization (FISH) was performed using a Cy3-labelled circKPNA2-specific probe (Table 1). After incubation with cells, nuclei were stained with DAPI. Observations were then recorded using a fluorescence microscope. Subsequently, nuclear and cytoplasmic fractions were isolated using a Cytoplasmic & Nuclear RNA Purification Kit (Norgen Biotek, Canada). Quantitative PCR(qPCR) was then performed to detect the distribution of circKPNA2 in the nuclear and cytoplasmic fractions. U6 and GAPDH were used as nuclear and cytoplasmic reference genes for normalization, respectively.

### RNase R resistance experiment

Equal amounts of total RNA were treated with RNase R (3 U/μg RNA) at 37°C for 30 minutes (RNase R group) or left untreated (mock group). qPCR was then performed to assess the resistance of circKPNA2 to RNase R digestion.

### CCK-8 assay

The Biotime® Cell Counting Kit-8 was used to detect HT29 cell proliferation. Cells were seeded at a density of 1 × 10^4^ cells per well, and 200 μL of cell suspension. After 12 hours of incubation to allow cell attachment, cell proliferation was measured at 24, 48, and 72 hours. At each time point, 20 μL of CCK-8 solution was added to each well, followed by incubation for 1 hour. Absorbance at 450 nm (A_450_) was measured using a microplate reader.

### Colony formation assay

Cells were seeded at 600 cells per well in 6-well plate. Once colonies exceeded 300 per well, the medium was removed. Cells were fixed with 4% paraformaldehyde for 5 minutes. Cells were stained with 0.5% crystal violet at room temperature for 10 minutes. Colonies were counted and images were captured using an Olympus optical microscope.

### Wound healing migration assay

HT29 cells were subjected to different treatments and prepared into a single-cell suspension (6 × 10^5^ cells/mL) using complete McCoy’s 5A medium. Then, 1 mL of cell suspension was added per well of a 6-well plate. After 12 hours of incubation at 37°C, a scratch was made in the cell monolayer using a sterile pipette tip. The initial wound area was recorded at 0 hours using a digital camera‑equipped inverted microscope. Additional images were captured at 24, 48, and 72 hours. Wound healing was quantified using ImageJ software. The percentage of wound closure was calculated as: [(wound area at 0 h − wound area at time t) / wound area at 0 h] × 100%.

### Cell invasion analysis

Cell invasion was assessed using Transwell chambers with 8‑μm pore size. Cells were passaged 2–3 times. Then, they were serum-starved for 24 hours prior to the assay. The Transwell chambers was pre-coated with Matrigel.Next, 200 μL of serum-starved cells, adjusted to 1.0 × 10^6^ cells/mL in serum-free medium, was added to the upper chamber, while the lower chamber received 500 μL of complete medium containing 10% foetal bovine serum (FBS). The setup was incubated at 37°C with 5% CO_2_ for 24 hours. After incubation, the chamber was washed twice with 1×PBS, fixed with 4% paraformaldehyde for 5 min, washed twice more, and stained with 0.1% crystal violet. Images of cells that had migrated through the Matrigel-coated membrane were capture, and ImageJ software was used to analyse the area covered by cells in each group.

### RNA pulldown and mass spectrometry analysis

The target sequence with T7 promoter was amplified and purified from the vector containing the target gene as a probe (Table 1). After biotin labelling, the RNA was bound to streptavidin magnetic beads, washed twice with wash buffer, and placed on a magnetic rack to remove the buffer. Biotin-labelled and denatured RNA was then added to the beads and incubated at 4°C for 2 h. After incubation, the magnetic bead-RNA-protein complex was collected by placing the tube on a magnetic rack; it was then centrifuged at low speed, and the supernatant was removed. The complex was washed three times with Wash Buffer. Cell lysate containing 1 mg of protein was added to the complex and incubated overnight at 4°C. The magnetic bead-RNA-protein complex was retrieved by placing the tube on a magnetic rack, centrifuged at low speed, and the supernatant was discarded, followed by washing three times with Wash Buffer. The supernatant was removed, and 40 μL Elution Buffer and 10 μL 5×SDS protein loading buffer were added; the mixture was then heated at 100°C for 10 min prior to electrophoresis. After electrophoresis, the gel was placed in an incubation box, and 100 mL PAGE gel fixing solution was added; the gel was incubated at room temperature for 40–60 min before silver staining and imaging. Finally, 100 mL ultrapure water was added to preserve the gel. After photographing, the gel bands of interest were excised and subjected to enzymatic digestion for mass spectrometry analysis. Mass spectrometry was performed using the Orbitrap Exploris 480 mass spectrometer coupled with the EASY-nLC 1200 liquid chromatography system under specified acquisition parameters. Data were searched against the UniProt Human Proteome Reference Database using the Andromeda algorithm in MaxQuant (v1.6.6).

### Western blot experiment

Total protein was extracted using a total protein extraction kit (Keygen BioTECH) and its concentration was determined. Twenty microlitres of total protein was mixed with SDS-PAGE loading buffer and heated at 100°C for 5 min. Subsequently, electrophoresis was performed using a 10% SDS-PAGE gel. After electrophoresis, the protein was transferred to a PVDF membrane, which was then blocked and washed three times with TBST. The membrane was incubated with primary antibody overnight at 4°C. After three washes with TBST, the membrane was incubated with horseradish peroxidase‑conjugated secondary antibody at room temperature for 1 hour. The membrane was washed three times with TBST. Chemiluminescent detection was performed using an ECL Western Blotting Analysis System kit according to the manufacturer’s instructions. Imaging was conducted using a gel imaging system. Densitometric analysis of the bands was performed using ImageJ 1.8.0 software. The antibodies used in the experiment included RIN1 (ab1389097, 1:1000), METTL3 (ab240595, 1:1000), ERK1/2 (ab184699, 1:1000), p-ERK1/2 (ab201015, 1:1000), Ras (ab108602, 1:1000), and p-Ras (ab151719, 1:1000), all from Abcam. GAPDH (60004–1-Ig, 1:5000) and Tubulin (66031–1-Ig, 1:5000) from Proteintech.

### meRIP-qPCR experiment

Total RNA was extracted using TRIzol. Genomic DNA was removed by DNase I digestion. Then, 5–20 μg of RNA was fragmented to lengths of 100–200 nucleotides using a Zn^2+^ -containing fragmentation buffer (94°C, 5 min) and purified for quality control. Protein A/G magnetic beads were first incubated with the anti-m^6^A antibody, or IgG control, at room temperature for 30 min to form antibody-bead complexes. Fragmented RNA was then added, and the mixture was incubated under rotation at 4°C for 4 h. The complex was washed sequentially with low-salt buffer (containing 0.05% NP-40) and high-salt buffer (containing 500 mM NaCl). RNA was eluted by incubation with an elution buffer containing 1% SDS at 55°C for 15 min. Input controls (1% of total RNA) were subjected to the same processing steps in parallel. RNA was extracted using phenol-chloroform, followed by ethanol precipitation for purification. cDNA was synthesized by reverse transcription, and SYBR Green qPCR was used to detect the target gene. The immunoprecipitation antibody used was anti-N6-methyladenosine (m^6^A) (ab284130, Abcam).

### Animal tumorigenesis experiment

All animal experiments were approved by the Institutional Animal Care and Use Committee of Ningxia Medical University (Ethics approval number: 2020–752). All procedures were performed in accordance with the guidelines of the Ningxia Medical University and the American Veterinary Medical Association (AVMA) Guidelines for the Euthanasia of Animals (2020 edition). Hangzhou Ziyuan Experimental Animal Technology Co., Ltd. Animal licence numbers: 20240518Abzz0105000589. For tumour xenograft experiments, mice were anaesthetised with intraperitoneal injection of ketamine (80 mg/kg) and xylazine (10 mg/kg) prior to subcutaneous injection of cancer cells. At the end of the experiment, mice were euthanized by cervical dislocation under isoflurane anaesthesia (induction 3%, maintenance 1.5% in 100% oxygen). All efforts were made to minimize suffering.

HT29 cells, METTL3 knockdown cells, and METTL3 knockdown cells overexpressing circKPNA2 were cultured and prepared. Six-week-old female immunodeficient NOD-SCID mice were used in this study. Mice were randomly assigned to experimental groups (6 per group, total 18) prior to tumour inoculation. The randomization sequence was generated a priori using a computer‑based random number generator (https://www.random.org).The sample size was chosen based on preliminary experiments showing a predicted difference in tumour volume of >30% between groups and was consistent with previous similar xenograft studies. All animal procedures and subsequent tumour measurements were performed by investigators blinded to group allocation. Mice were housed in individually ventilated cages (IVC) under specific pathogen‑free conditions, with a maximum of 5 mice per cage. The environment was maintained at a temperature of 22 ± 2°C, relative humidity of 50 ± 10%, and a 12‑h light/dark cycle (lights on from 8:00 to 20:00). Sterilized pellet food and autoclaved tap water were provided ad libitum. Bedding (corncob) was changed once weekly. Mice were acclimated to the housing conditions for one week before the start of the experiment. No mice were excluded due to death or tumour ulceration during the experiment. Mice were acclimated for one week. Exponentially growing HT29 cells were collected and adjusted to a concentration of 1 × 10^7^ cells per 100 μL per mouse. A ruler was placed alongside tumours for photographic documentation, and tumour size was measured once per week in animal laboratory. Tumour volume was calculated using the formula: 0.5 × long diameter × (short diameter)^2^ (mm^3^).

### Statistical analysis

Differences between paired colorectal cancer and adjacent normal tissues were analysed using a paired Student’s t-test. Independent-sample t-tests were used for comparisons between two groups, and one-way ANOVA was used for multiple group comparisons. Normality: Shapiro‑Wilk test; homogeneity: Levene’s test; sphericity: Mauchly’s test with Greenhouse-Geisser correction if violated. If assumptions violated, non‑parametric alternatives (Kruskal – Wallis) or data transformation were used. Data were analysed using SPSS 23.0, and graphing was performed using GraphPad Prism 9. *p*-value < 0.05 was considered statistically significant.

## Results

### CircKPNA2 is upregulated in colorectal cancer

**Figure 1.**
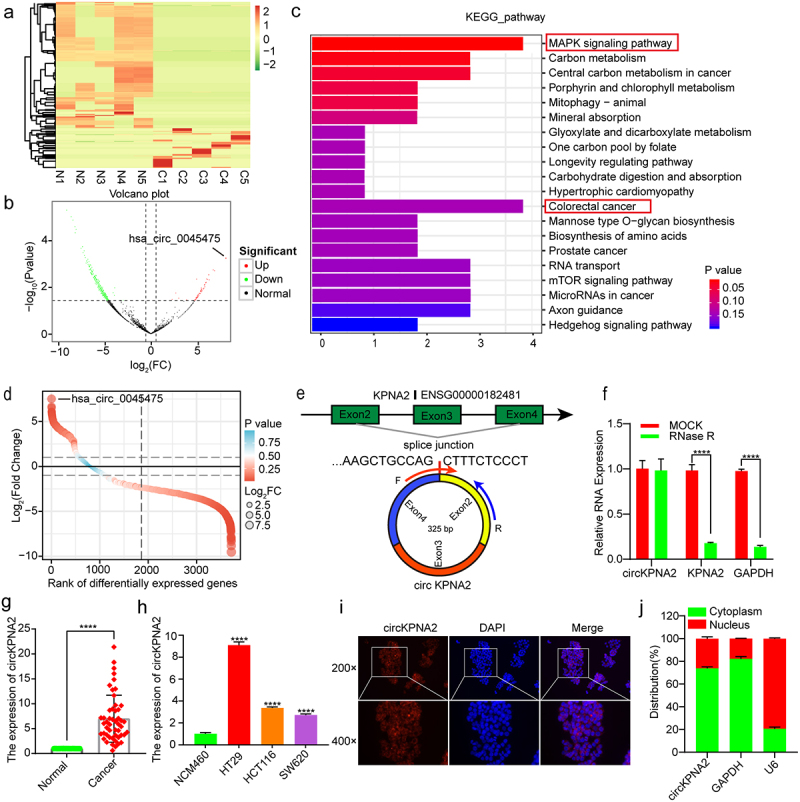
Sequencing analysis of circRNA and validation of circKPNA2 expression abundance and distribution in CRC. a. Heatmap depicting the expression profiles of differentially expressed circRnas in normal(N1-N5) and CRC(C1-C5) tissues samples. b. Volcano plot of differentially expressed circRnas; green, red and black points represent downregulated, upregulated, and non-differentially expressed circRnas, respectively. c. KEGG pathway enrichment analysis of differentially expressed circRnas; the color scale indicating the *p*-value. d. Ranking of differentially expressed circRnas based on log_2_(FC) and *p*-value. e. Schematic representation of circKPNA2 biogenesis derived from the KPNA2 gene. f. Relative expression of circKPNA2, linear KPNA2, and GAPDH (as control) following RNase R treatment. g. Expression levels of circKPNA2 in 50 paired normal and CRC tissue samples. h. Expression of circKPNA2 in normal intestinal epithelial cells (NCM460) and CRC cell lines (HT29, HCT116, SW620). i. Fluorescence in situ hybridization (FISH) showing the subcellular localization of circKPNA2 (red) and DAPI (blue). j. Quantification of the subcellular distribution of circKPNA2 and GAPDH mRNA. **** denotes *p < 0.001*.

To identify circRNAs abnormally expressed in CRC, we performed whole-transcriptome RNA-seq analysis on cancer and normal tissues. The heatmap showed significant differences in gene expression clustering between the two groups of samples (Figure 1(a)), suggesting that circRNAs might play a role in the regulation of CRC. Further analysis using a volcano plot identified differentially expressed circRNAs with |log_2_ fold change| >1 and adjusted *p*-value < 0.05, among which hsa_circ_0045475 (circKPNA2) exhibited the most significant upregulation (Figure 1b). KEGG pathway enrichment analysis indicated that the host genes of differentially expressed circRNAs were significantly enriched in the MAPK signalling pathway and the CRC pathway (Figure 1c), suggesting that circKPNA2 May promote tumorigenesis by regulating these pathways. Based on differential gene ranking analysis, circKPNA2 showed significant upregulation among the differential genes (Figure 1d). CircKPNA2 is generated by back-splicing of exons from KPNA2 (Figure 1e) and, unlike linear KPNA2, is resistant to RNase R, confirming its circular RNA nature (Figure 1f). Clinical sample and cell line analyses showed that circKPNA2 was significantly overexpressed in cancer tissues and in the HT29, HCT116 and SW620 cell lines compared with normal tissues and the normal intestinal epithelial cell line NCM460 (Figure 1(g, h)). Subcellular localization experiments indicated that circKPNA2 was mainly distributed in the cytoplasm (Figure 1(i, j)), similar to the distribution pattern of GAPDH, while U6 was primarily localized in the nucleus, further clarifying its subcellular distribution. Furthermore, the significant overexpression of circKPNA2 in clinical CRC specimens and its cytoplasmic localization suggest its potential role as a novel diagnostic biomarker or therapeutic target for CRC.

### CircKPNA2 promotes CRC progression by enhancing cell proliferation, invasion, and migration

**Figure 2.**
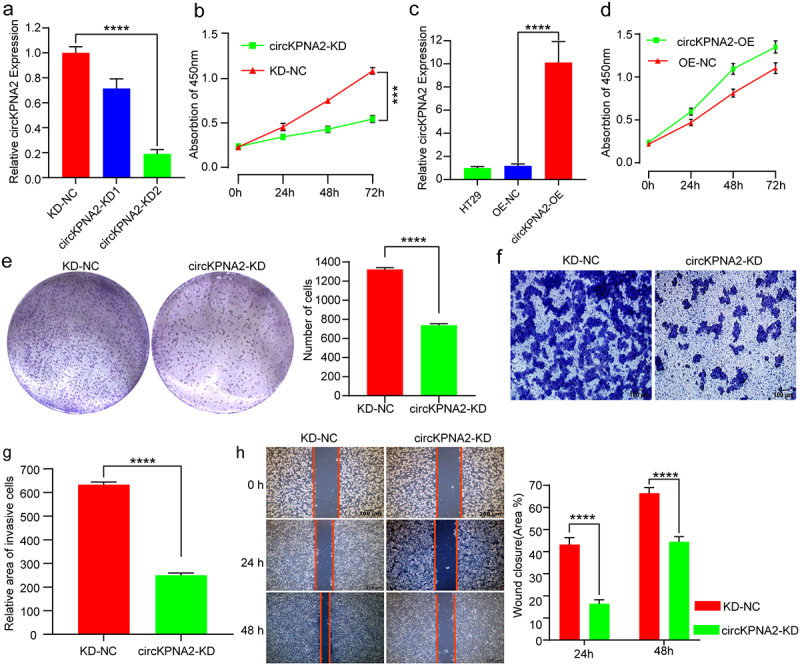
circKPNA2 promotes the proliferation, invasion, and migration of CRC cells. a. qPCR analysis of circKPNA2 expression in HT29 cells after transfection with circKPNA2 knockdown constructs KD1, KD2, or negative control (KD-NC). b. CCK-8 assay detect the proliferation of HT29 cells transfected with circKPNA2 knockdown constructs or KD-NC at 0, 24, 48, and 72 hours. c. qPCR analysis of circPNA2 expression in HT29 cells transfection with circKPNA2 overexpression (OE) construct. d. CCK-8 assay detect the proliferation of HT29 cells transfected with circKPNA2 OE at 0, 24, 48, and 72 hours. e. Colony formation assay evaluating the clonogenicity of HT29 cells after circKPNA2 knockdown, the histogram quantifies the number of colonies. f. Transwell invasion assay of HT29 cells transfected with circKPNA2-KD. g. Quantification of the relative area of invaded cells from the Transwell assay. h. Wound healing assay was performed to examine the migration of HT29 cells after circKPNA2-KD at 0, 24, and 48 hours, the histogram shows the wound closure area. **** denotes *p < 0.001*.

To explore the biological function of circKPNA2, we designed siRNA-mediated knockdown (KD) and overexpression (OE) vectors. These vectors were then transfected into the CRC cell line HT29. qPCR results showed that circKPNA2 expression was significantly reduced in the KD group compared with the negative control group (KD-NC), indicating effective siRNA silencing (Figure 2(a)). The expression level of circKPNA2 in the overexpression group (OE) was significantly higher than that in the control group (OE-NC), confirming the stability and efficiency of the overexpression system. Using CCK-8 assays to measure cell proliferation, we found that knockdown of circKPNA2 significantly reduced the optical density (OD) at 450 nm at 24 h, 48 h, and 72 h compared to the KD-NC control group, indicating inhibited cell growth (Figure 2(b)). In contrast, no statistically significant difference was observed between the OE group and the OE‑NC group (Figure 2(c, d)). Furthermore, colony formation assays confirmed that the number of colonies in the circKPNA2-KD group was significantly reduced compared to the KD-NC group, suggesting that circKPNA2 contributes to maintaining cancer cell proliferation (Figure 2(e)). Transwell invasion assays showed that the number of cells that migrated through the membrane in the circKPNA2-KD group was significantly reduced compared to the KD-NC group, indicating that circKPNA2 promotes the invasion of CRC cells (Figure 2(f, g)). Wound healing assay results demonstrated that the wound closure area in the circKPNA2-KD group at 24 h, 48 h, and 72 h was significantly lower than that of the KD-NC group, indicating that loss of circKPNA2 delays cell migration (Figure 2(h)). The lack of statistically significant effect on HT29 cell proliferation following circKPNA2 overexpression can be attributed to two reasons: (1) the endogenous circKPNA2 expression level in HT29 cells is already high, so the incremental effect of exogenous overexpression is not significant in short-term proliferation assays; (2) CCK-8 measures overall metabolic activity, whereas colony formation reflects long-term clonogenic potential, which is more sensitive to circKPNA2 modulation. Therefore, circKPNA2 promotes malignant progression in CRC by enhancing cell proliferation, invasion, and migration, providing experimental evidence to elucidate its oncogenic mechanism and inform targeted therapeutic strategies.

### CircKPNA2 promotes colorectal cancer progression by regulating the Ras pathway through binding to RIN1

**Figure 3.**
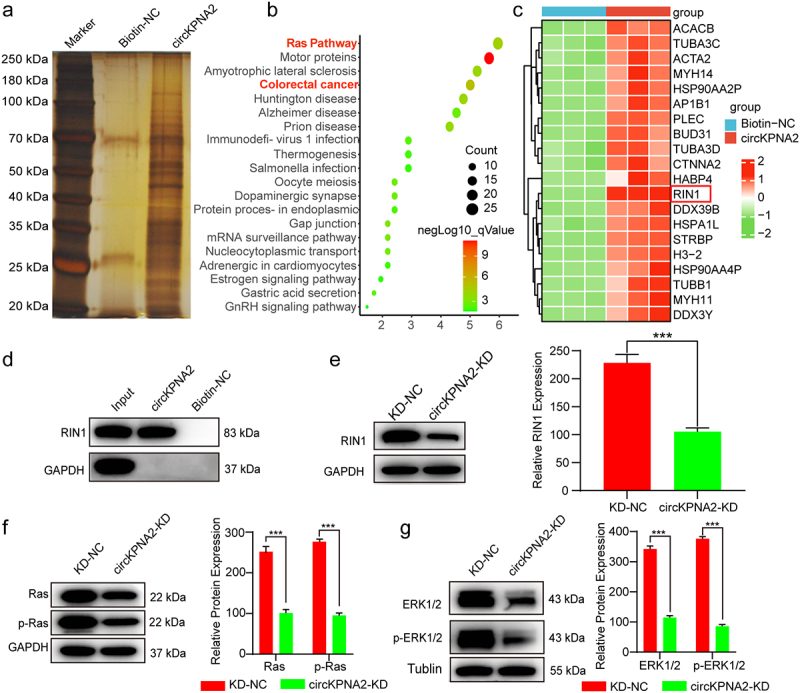
Mechanistic insights into the function of circKPNA2. a. Silver staining of proteins pulled down by biotin-labeled circKPNA2 and biotin-NC (negative control). b. KEGG pathway enrichment analysis of proteins bound to circKPNA2. c. Top 20 candidate proteins interacting with circKPNA2 identified by mass spectrometry. d.Western blot analysis of RIN1 protein in Input, circKPNA2 pulldown, and biotin-NC groups, confirming the interaction between circKPNA2 and RIN1. e. Western blot (left) and qPCR(right) of RIN1 expression in circKPNA2-KD and KD-NC groups. f. Western blot (left) and qPCR(right) of Ras and p-Ras protein levels in circKPNA2-KD and KD-NC groups. g. Western blot (left) and qPCR(right) of ERK1/2 and p-ERK1/2 expression in circKPNA2-KD and KD-NC groups. *** denotes *p < 0.01*.

We used biotin-labelled circKPNA2 in RNA pulldown experiments, comparing it with biotin-labelled negative control RNA (Biotin-NC). Western blot analysis initially detected several proteins potentially interacting with circKPNA2, as indicated by distinct molecular weight bands (Figure 3(a)). The top 20 proteins most significantly enriched in the mass spectrometry results are listed (Figure 3(c)), with the key protein RIN1 highlighted in this study. Based on preliminary screening results and subsequent analysis, RNA binding protein immunoprecipitation (RIP) experiments were performed. The results show that RIN1 protein is specifically enriched in the total protein bound to circKPNA2 (Input group) compared with the control group, confirming the specific binding between circKPNA2 and RIN1 (Figure 3(d)). To explore the functional impact of circKPNA2 on RIN1, we knocked down circKPNA2 in CRC cells (circKPNA2-KD). Western blot analysis reveals that RIN1 protein expression is significantly reduced in the circKPNA2-KD group compared to the KD-NC group, indicating that circKPNA2 positively regulates RIN1 expression (Figure 3(e)).

Proteins associated with circKPNA2 were analysed by mass spectrometry. Moreover, the results show that genes related to circKPNA2 are significantly enriched in the Ras signaling pathway and other related signalling pathways (Figure 3(b)). Furthermore, validation of the Ras pathway reveals the presence of key Ras pathway proteins in circKPNA2-KD cells. Western blot and quantitative analyses show that total Ras and phosphorylated Ras (p-Ras) protein levels are significantly reduced in the circKPNA2-KD group compared to the KD-NC group (Figure 3(f)), Indicating that knocking down circKPNA2 inhibits the activation of the Ras pathway. Additionally, analysis of the key Ras pathway protein ERK1/2 revealed a significant decrease in both total ERK1/2 and its phosphorylated form (p-ERK1/2) in the circKPNA2-KD group relative to the KD-NC group (Figure 3(g)). Taken together, this study suggests that circKPNA2 can bind to RIN1 and influence Ras pathway activation by regulating RIN1 expression, providing evidence for the role of circKPNA2 in related biological processes. These findings indicate that circKPNA2 May participate in regulation through the Ras pathway, with its function depending on its interaction with RIN1 and subsequent effects on downstream pathway proteins.

### CircKPNA2 deficiency delays colorectal cancer progression by inhibiting the RIN1/Ras axis

**Figure 4.**
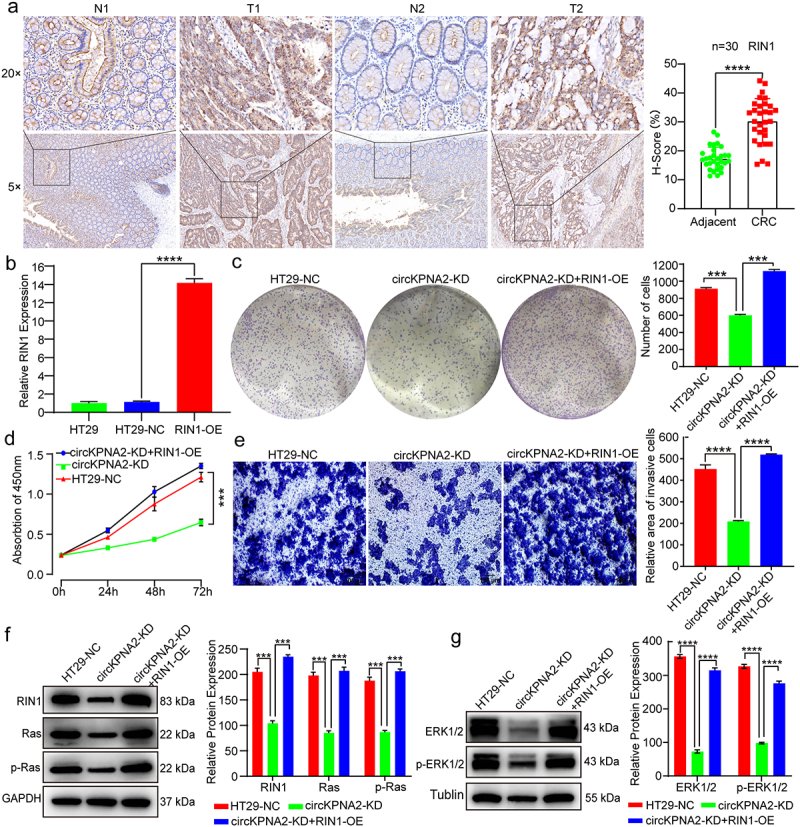
RIN1 promotes the progression of CRC by activating the Ras/ERK signaling pathway. a. Immunohistochemical staining (×20, ×5 magnification) and H-score quantification of RIN1 in CRC (t) and adjacent (N) tissues. b. qRT‒PCR analysis of RIN1 expression in HT29 cells transfected with negative control (NC) or RIN1 overexpression plasmid (RIN1-OE). c, d. Colony formation assay and CCK-8 assay measured the proliferation of HT29-NC, circKPNA2-KD, and circKPNA2-KD + RIN1-OE group. e. Transwell invasion assay of HT29-NC, circKPNA2-KD, and circKPNA2-KD + RIN1-OE group. f, g. Western blot analysis of RIN1, Ras, p-Ras, ERK1/2, and p-ERK1/2 expression in HT29-NC, circKPNA2-KD, and circKPNA2-KD + RIN1-OE group. *** denotes *p < 0.01*. **** denotes *p < 0.001*.

To explore the role of RIN1 in CRC, we first measured its expression in CRC tissues and paired adjacent normal tissues. Immunohistochemistry was used to detect RIN1 expression. The results showed stronger RIN1 protein staining in CRC tissues, whereas adjacent normal tissues exhibited weaker staining. Further quantification using the H-score indicated that RIN1 expression was significantly higher in CRC tissues than in adjacent normal tissues, suggesting that RIN1 May have an oncogenic role in CRC progression (Figure 4(a)). To clarify the biological function of RIN1, we knocked down circKPNA2 (circKPNA2-KD) in the HT29 cell line. We then established a model of circKPNA2-KD combined with RIN1 overexpression (circKPNA2-KD + RIN1-OE) to conduct functional experiments. The colony formation assay showed that the number of colonies significantly decreased in the circKPNA2-KD group compared with the control (HT29-NC), while RIN1 overexpression (circKPNA2-KD + RIN1-OE) significantly restored colony numbers compared to circKPNA2-KD alone (Figure 4(c)). The CCK-8 proliferation experiment further confirmed that the proliferation rate in the circKPNA2-KD group was significantly reduced, whereas the proliferation rate in the circKPNA2-KD + RIN1-OE group significantly increased compared with the circKPNA2-KD group (Figure 4(d)). These rescue experiments showed that RIN1 overexpression restored both colony formation and proliferation that had been reduced by circKPNA2-KD.

Transwell invasion assay results showed that the number of invasive cells that migrated through the membrane in the circKPNA2-KD group was significantly reduced compared to the control group (Figure 4(e)). However, RIN1 overexpression effectively reversed the inhibitory effect of circKPNA2-KD on cell invasion. Therefore, RIN1 significantly promotes the proliferation and invasion of CRC cells, and its expression is positively regulated by circKPNA2.

To elucidate the molecular mechanism by which RIN1 regulates malignant behaviour in CRC cells, we detected the expression changes of key molecules in the Ras signaling pathway using Western blot analysis. The results showed that, compared to the control group, the protein levels of RIN1, p-Ras (phosphorylated Ras), and p-ERK1/2 (phosphorylated ERK1/2) were significantly downregulated in the circKPNA2-KD group. After RIN1 overexpression (circKPNA2-KD + RIN1-OE group), the levels of p-Ras and p-ERK1/2 significantly recovered, indicating that RIN1 knockdown inhibited the activation of this pathway (Figure 4(f, g)). Our results demonstrate that RIN1 is highly expressed in CRC tissues and promotes malignant behaviours, such as proliferation and invasion of CRC cells, by activating the Ras signalling pathway. This finding provides new insights into CRC development. It also offers experimental evidence to further elucidate CRC pathogenesis and to develop intervention strategies targeting RIN1.

### METTL3 mediating m^6^a modification regulates circKPNA2 expression

**Figure 5.**
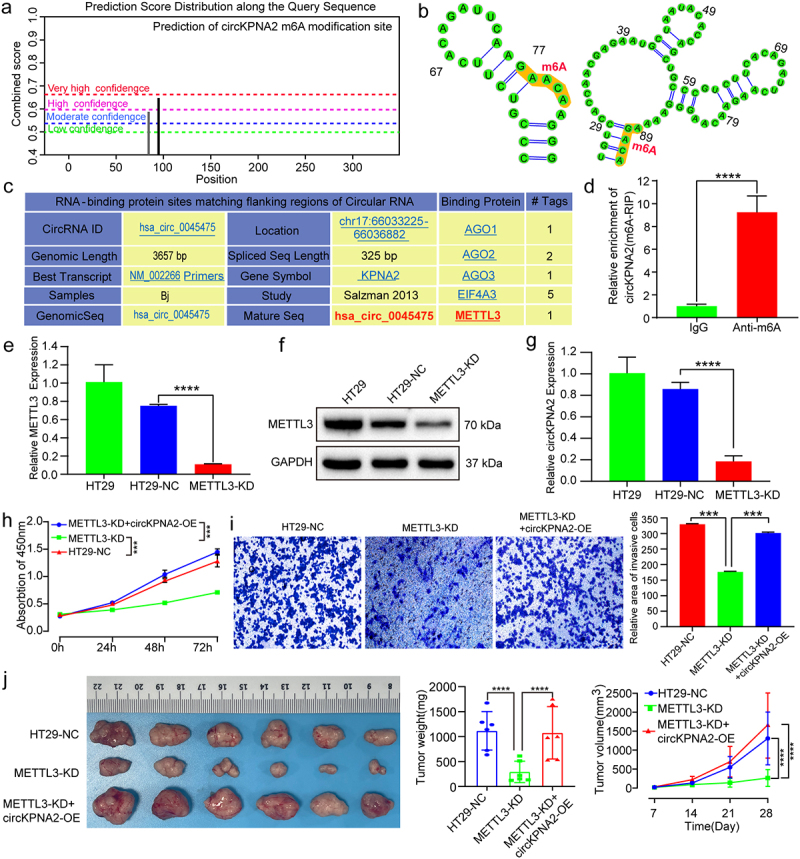
circKPNA2 is modified by m^6^A in a METTL3-dependent manner and promotes CRC cell proliferation and tumorigenesis in vivo. a. Prediction of m^6^A modification sites on circKPNA2 by the SRAMP bioinformatics tool. b. Schematic diagram of the circKPNA2 secondary structure with predicted m^6^A sites indicated. c. Potential RNA-binding proteins targeting the flanking sequences of circKPNA2. d. RIP-qPCR analysis showing the enrichment of circKPNA2 by an anti-m^6^A antibody compared to IgG control. e. qPCR analysis of METTL3 expression in HT29, HT29-NC, and METTL3-KD cells. f. Western blot analysis of METTL3 expression levels in HT29, HT29-NC, and METTL3-KD cells. g. qPCR analysis of circKPNA2 expression in HT29, HT29-NC, and METTL3-KD cells. h. CCK-8 assay showing cell proliferation of HT29-NC, METTL3-KD, and METTL3-KD + circKPNA2-OE cells. i. Transwell assay showing the migration of HT29-NC, METTL3-KD, and METTL3-KD + circKPNA2-OE cells. j. Xenograft tumor growth in nude mice injected with HT29-NC, METTL3-KD, and METTL3-KD + circKPNA2-OE cells (left); tumor volume and weight quantification (right). *** denotes *p < 0.01*. **** denotes *p < 0.001*.

We performed bioinformatics analysis of the circKPNA2 nucleotide sequence. Using the SRAMP database, we predicted potential m^6^A modification sites. The predicted scores showed multiple high-confidence potential m^6^A modification regions within the circKPNA2 sequence (Figure 5(a)), helping to identify specific modification sites for further investigation. We used an RNA secondary structure prediction model to predict the secondary structure of circKPNA2 and locate the predicted m^6^A modification sites (Figure 5b), which facilitated understanding of how m^6^A modification may affect circKPNA2’s spatial conformation and function. To identify m^6^A-related protein that interact with circKPNA2, we searched the CircInteractome database using its sequence and found several potential interacting RNA-binding proteins (RBPs), including AGO1, AGO2, and the m^6^A methyltransferase METTL3 (Figure 5(c)). To determine whether circKPNA2 undergoes m^6^A modification and to quantify its level, we performed m^6^ A immunoprecipitation (m^6^A-RIP) experiments. We immunoprecipitated m^6^A-modified RNA from total RNA extracted from whole HT29 cells using an anti-m^6^A antibody, with control IgG serving as the negative control. Following immunoprecipitation, we measured the relative enrichment of circKPNA2 in the immunoprecipitate by qRT-PCR, normalized to input. The results demonstrated that circKPNA2 was significantly enriched in the m^6^A-RIP samples, consistent with database predictions (Figure 5(d)).

To investigate METTL3 regulation of circKPNA2, we knocked down METTL3 in the HT29 CRC cell line, allowing us to examine METTL3’s regulatory role on circKPNA2. qPCR and Western blot analyses showed that, compared with control cells (HT29-NC), METTL3 knockdown (METTL3-KD) significantly reduced METTL3 expression at both the mRNA and protein levels, establishing a stable knockdown model (Figure 5(e, f)). After METTL3-KD, we measured circKPNA2 expression and found it was significantly downregulated (Figure 5(g)). These results suggest that METTL3 regulates circKPNA2 through its m^6^A methyltransferase activity, establishing a preliminary ‘METTL3-m^6^A-circKPNA2’ regulatory axis supported by our experimental evidence.

### METTL3 regulates circKPNA2 expression, promoting CRC proliferation and metastasis

Cell function experiments showed that METTL3-KD significantly inhibited the proliferation (Figure 5(h)) and migration (Figure 5(i)) of HT29 cells. Importantly, overexpression of circKPNA2 in the METTL3 knockdown context (METTL3-KD + circKPNA2-OE) partially restored the suppressed proliferation and migration of HT29 cells (Figure 5(h, i)). This suggests that circKPNA2 is a key downstream effector of METTL3, regulating cell proliferation and migration. In vivo mouse xenograft tumour models further supported these findings. Tumour growth was significantly slower in the METTL3‑KD group than in the control group (HT29‑NC). Specifically, tumour volume was 265.7 ± 202.9 mm^3^ in the METTL3‑KD group versus 1308.5 ± 635.8 mm^3^ in the control group (mean ± SEM, *p* < 0.001), and tumour weight was 292.2 ± 195.4 mg versus 1116.4 ± 352.0 mg (*p* < 0.001), indicating a marked reduction. However, overexpression of circKPNA2 in METTL3‑KD cells (METTL3‑KD + circKPNA2‑OE) significantly reversed this inhibition. In the METTL3‑KD + circKPNA2‑OE group, tumour volume increased to 1668.6 ± 801.0 mm^3^ (vs. 265.7 ± 202.9 mm^3^ in METTL3‑KD, *p* < 0.001), and tumour weight to 1076.5 ± 379.3 mg (vs. 292.2 ± 195.4 mg, *p* < 0.001) (Figure 5j). These results confirm that circKPNA2 promotes tumorigenesis in vivo and that METTL3 regulates its function.This study identifies specific m^6^A modification sites on circKPNA2 and its interacting proteins, confirming that METTL3 regulates circKPNA2 expression by modulating its m^6^A levels. The study also clarifies the key role of circKPNA2 in promoting CRC proliferation, migration, and tumorigenesis. These findings provide important insights into the epigenetic regulation of circRNA and its molecular mechanisms in cancer development.

## Discussion

CRC is a common malignancy worldwide, with incidence influenced by genetic, dietary, lifestyle, and environmental factors [12–14]. Its pathogenesis involves gene mutations, epigenetic changes, and tumour microenvironment alterations [15,16]. Among these, circRNAs play a key role by regulating proliferation, invasion, and metastasis [17]. However, the functions and mechanisms of many circRNAs remain largely unexplored.

This study identified circKPNA2 as a significantly upregulated circRNA in CRC. Using RNA sequencing, functional assays, RNA pull‑down, and mass spectrometry, we investigated its effects on CRC cell proliferation, invasion, and migration, as well as its regulation of RIN1 and the Ras signalling pathway. To our knowledge, the role of circKPNA2 in CRC has not been previously reported. Notably, its linear host gene KPNA2 is highly expressed in CRC and is an independent poor prognostic factor [18]; it also interacts with Wip1 to regulate the AKT/GSK‑3β pathway in a p53‑dependent manner^2^.

We found that circKPNA2 promotes CRC proliferation and metastasis by upregulating RIN1 and activating the Ras pathway. RIN1, a multifunctional signalling protein, is involved in TGF‑β‑induced fibroblast migration; its phosphorylation by PKA inhibits Ras/Raf/ERK signalling and reduces migration and fibrosis [19]. In CRC, C20orf24 recruits RIN1, and activated Rab5 promotes Ras activation, driving sustained MAPK/ERK signalling [20]. Aberrant PI3K/AKT/mTOR activation drives CRC progression, and liraglutide inhibits this pathway to suppress proliferation and invasion while promoting apoptosis [21]. Linc01615, an oncogene in CRC, is negatively regulated by miR-491-5p to modulate proliferation, apoptosis, and migration [22]. Novel oncolytic adenoviruses ADVNE and ADVPPE induce pyroptosis and enhance antitumor immunity [23]. Machine learning is increasingly applied in CRC microbiome and diagnostic research [24]. Our study found that RIN1 is highly expressed in CRC and is regulated by circKPNA2 at the expression level. When circKPNA2 is knocked down, Ras signalling pathway activity is inhibited. This mechanism provides new insights into CRC pathogenesis and potential therapeutic targets, particularly for drugs aimed at the Ras signalling pathway, whose activation is linked to the development of various tumours [25–28]. Thus, circKPNA2 May play a key role in cancer initiation and progression as a regulator of the Ras signalling pathway, providing new directions for future therapeutic development.

The METTL3‑circKPNA2‑RIN1‑Ras axis is novel in CRC. First, while most m^6^A studies focus on circRNA cyclization or nuclear export, we show that METTL3 directly upregulates circKPNA2 via m^6^A modification. Second, unlike known Ras pathway mutations, this axis represents a non‑mutation‑dependent mode of activation via circRNA‑mediated RIN1 regulation. Third, it links epitranscriptomic regulation to malignant phenotypes, providing an intervention node targeting METTL3 or circKPNA2 itself.

Experiments on CRC cell function showed that knocking down circKPNA2 significantly inhibited both cell proliferation and migration. These results alter our understanding of tumour cells’ adaptive mechanisms in different environments and support the importance of circRNA in cancer biology. Previous studies show that the regulatory role of circRNA in cell proliferation and migration is linked to the malignant characteristics of cancer [29,30]. We also found m^6^A modification sites on the circular structure of circKPNA2. Moreover, we observed that circKPNA2 expression is regulated by the methyltransferase METTL3, providing new insights into the regulatory mechanisms governing circRNA expression. Studies show that m^6^A modification of circRNA is crucial for its expression and function [5,31]. For example, in breast cancer, m^6^A-modified circATAD2 enhances PD-L1 mRNA stability via the m^6^A reader IGF2BP3, thereby inhibiting anti-tumour immune surveillance by CD8^+^ T cells and highlighting its role in immune evasion [32]. In CRC, WTAP, a component of the m^6^A methyltransferase complex, is significantly highly expressed and can activate the MAPK pathway via the WTAP/YTHDC1/VEGFA axis, thereby promoting CRC proliferation, migration, invasion, and angiogenesis, positioning it as a potential biomarker [33]. m^6^A also interacts with programmed cell death to influence therapy resistance [34]. In pancreatic neuroendocrine tumours, ALKBH5 regulates lipid metabolism via the FABP5/mTOR axis [35]. Furthermore, m^6^A RNA modification and its writer and reader proteins are also aberrantly regulated in non-tumour diseases such as epilepsy, m^6^A regulators affect neuronal excitability and may serve as biomarkers [36]. Environmental factors such as PM2.5 can also upregulate piR-27222, reducing Casp8 transcript stability via the eIF4B/WTAP/m6A axis and inhibiting cellular PANoptosis, thereby promoting lung cancer development, revealing a novel epigenetic mechanism of environmental exposure-induced tumorigenesis [37]. Overall, m^6^A modification forms complex feedback loops with non‑coding RNAs, influencing the tumour microenvironment and therapy response [38]. Despite these findings, numerous aspects of circRNA m^6^A modification, including its regulatory mechanisms and functional implications, remain unexplored.

This study has limitations. The small sample size may not represent the entire CRC population, and the findings lack clinical validation. Moreover, although we confirmed circKPNA2‑RIN1 binding, we did not map the specific binding regions due to technical concerns about disrupting the circular structure, which could introduce false positives. Therefore, future studies should expand the sample size and conduct clinical validation to support these results and enhance their clinical value.

In summary, this study demonstrates significant circKPNA2 upregulation in CRC and its key role in promoting tumour progression via the RIN1 and Ras signalling pathways. Additionally, METTL3-mediated regulation of circKPNA2 expression provides new mechanistic insight. These findings offer important evidence that improves our understanding of colorectal cancer’s molecular mechanisms and aids in developing targeted therapies. Future research should further explore circKPNA2’s clinical application as a potential therapeutic target to advance CRC treatment.

## Supplementary Material

supplementary figure1.tif

## Data Availability

The RNA seq data or analysed during the current study are not publicly available due to research needs, it is temporarily inconvenient to disclose, but are available from the corresponding author on reasonable request. We can ensure the integrity of all data.
