# Supplementary figures and images for: m6A methylation of circKPNA2 promotes colorectal carcinogenesis by activating the RIN1-Ras pathway
Source: Epigenetics. 2026 Jun 19;21(1):2672219. doi: 10.1080/15592294.2026.2672219 (PMC13285648; doi:10.1080/15592294.2026.2672219)

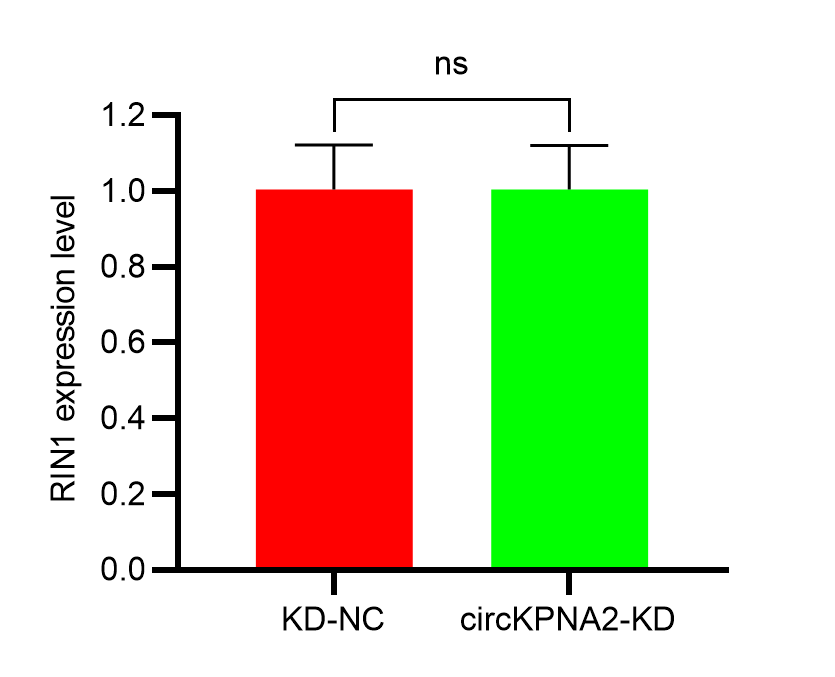

Supplement: supplementary figure1.tif [file KEPI_A_2672219_SM6691.tif]
